# Updated genetic background of generalized pustular psoriasis as an autoinflammatory keratinization disease

**DOI:** 10.1111/1346-8138.17585

**Published:** 2024-12-19

**Authors:** Masashi Akiyama

**Affiliations:** ^1^ Department of Dermatology Nagoya University Graduate School of Medicine Nagoya Japan

**Keywords:** autoinflammation, CARD14, IL‐36, *MEFV*, pyrin

## Abstract

Generalized pustular psoriasis (GPP) is a severe autoinflammatory keratinization disease (AiKD) characterized by acute flares of widespread sterile pustules and high fever. GPP is potentially life‐threatening. Recently clarified genetic predisposing factors for GPP suggest that the excessive activation of innate immune pathways in the skin, including of interleukin (IL)‐1 and IL‐36 signaling, plays a significant role in the GPP pathogenesis. *IL36RN*, *CARD14*, *AP1S3*, *MPO*, *SERPINA3*, *BTN3A3*, and *MEFV* have been identified as GPP‐related genes. The pathogenesis of GPP provoked by variants in these seven genes is tightly associated with the excessive activation of innate immune pathways and the resulting autoinflammation in the skin. Various biologics, including inhibitors for the tumor necrosis factor, IL‐17, and IL‐23 pathways, are used as treatments for GPP. The new understanding of the genetic background of GPP, mentioned above, indicates that the genetic predisposing factors are predominantly related to the excessive activation of innate immunity and autoinflammation. In this context, inhibitors of inflammatory signaling, including of the IL‐1 and IL‐36 pathways, have been used in clinical practice and investigated as potential future therapies.

## 1 INTRODUCTION

Generalized pustular psoriasis (GPP) is known as the most severe subtype of pustular psoriasis. GPP typically shows diffuse or widespread multiple erythematous lesions with sterile pustules, general malaise, and high fever.^1^ It frequently recurs throughout life and can be fatal.

In 2011, variants in *IL36RN*, which encodes the interleukin (IL)‐36 receptor antagonist (IL36Ra), were reported to be a genetic cause of GPP.^2^, ^3^ Impetigo herpetiformis (IH) is regarded as a pregnancy‐induced GPP, and a number of IH cases have been reported to have *IL36RN* variants.^4^, ^5^ Later, variants in *CARD14*, *AP1S3*, *MPO*, and *SERPINA3* were reported to confer susceptibility to GPP.^6^, ^7^, ^8^, ^9^, ^10^, ^11^ Recently, variants in *BTN3A3* ^12^ and *MEFV* ^13^ were demonstrated to be GPP‐related variants. GPP‐related variants in all seven genes are thought to induce the excessive activation of innate immunity and to bring about autoinflammation in the skin.

Inflammation induced by variants in innate immune pathway‐related genes in the skin sometimes give rise to inflammatory keratinization diseases, and such diseases that have a mainly genetic autoinflammatory pathogenesis are named autoinflammatory keratinization diseases (AiKDs).^14^, ^15^ Common phenotypes of AiKDs are hyperkeratotic lesions with inflammation, although the clinical manifestations are diverse.^16^

The recent elucidation of the genetic backgrounds and pathogenetic mechanisms of GPP has led us to the concept of GPP being provoked mainly by autoinflammation. In light of this, GPP is considered to be a clinical entity that is representative of AiKDs.^14^, ^15^

This review summarizes current knowledge on the genetic background and pathogenesis of GPP and provides prospects for new treatments that target molecules and inflammatory pathways that are thought to play crucial roles in the pathogenesis of GPP as an AiKD.

## 2 UPDATED GENETIC PREDISPOSING FACTORS FOR GPP

**FIGURE 1:**
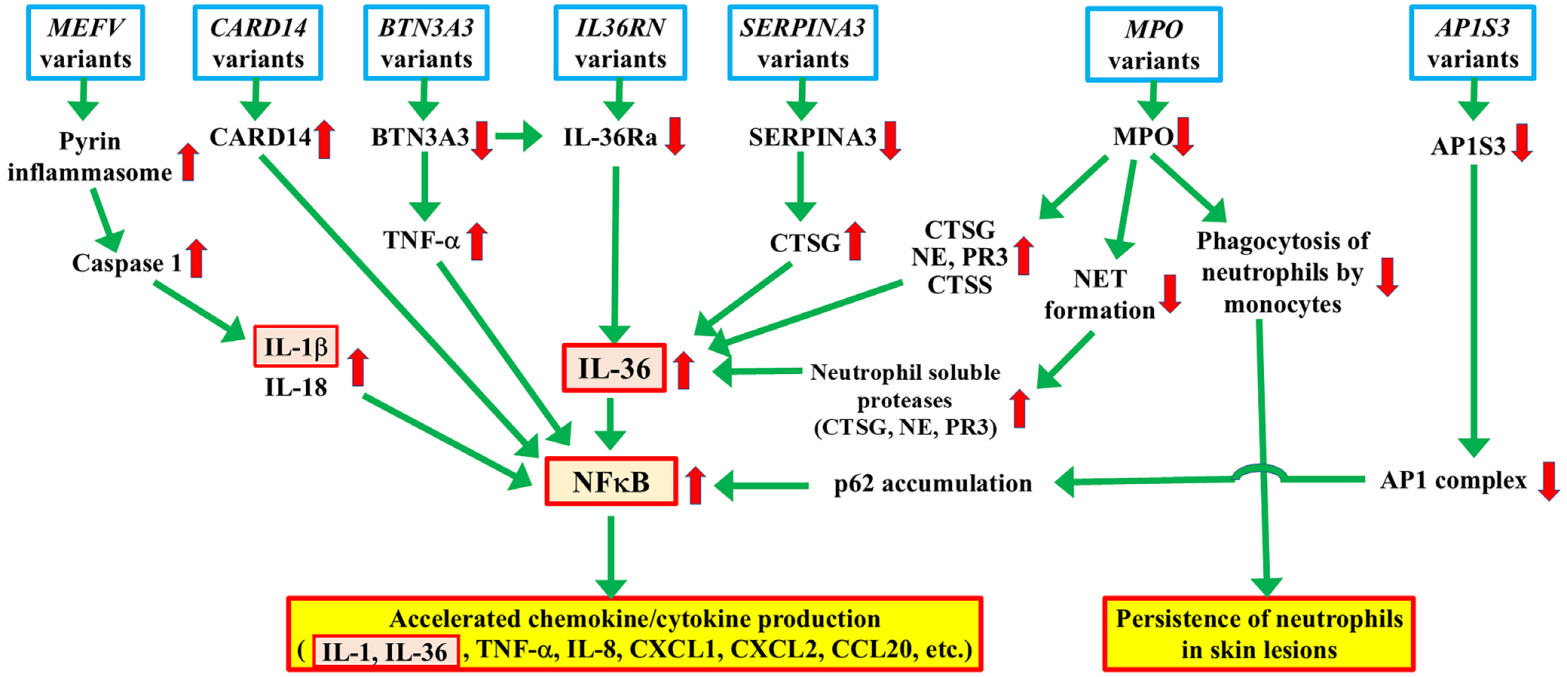
Genetic risk factors for generalized pustular psoriasis (GPP) and their downstream inflammation pathways, which are mainly associated with autoinflammation. Disease‐associated inflammation pathways and signaling in GPP patients carrying variants in genes encoding seven disease‐related molecules (IL‐36Ra, CARD14, AP1S3, SERPINA3, MPO, BTN3A3, and pyrin) are illustrated. Excessive activation of the IL‐36 pathway and NFκB plays a central role in the pathogenesis of GPP. The excessive activation of IL‐36 pathways resulting from IL‐36Ra deficiency and gain‐of‐function variants of *CARD14* upregulate NFκB activity. p62 accumulation resulting from impaired autophagy caused by AP1S3 deficiency also upregulates NFκB activity. Accelerated NFκB activity leads to the excessive secretion of chemokines/cytokines, such as TNF‐α, IL‐1, IL‐36, IL‐8, CXCL1, CXCL2, and CCL20, from keratinocytes. These chemokines/cytokines activate neutrophils/dendritic cells. Loss‐of‐function variants in *SERPINA3* result in the deficient inhibition of cathepsin G (CTSG), leading to the excessive activation of IL‐36 precursors and upregulated IL‐36 signaling. In addition, loss‐of‐function variants of *MPO* result in the defective inhibition of neutrophil proteases and result in increased amounts of soluble proteases via defective neutrophil extracellular trap (NET) formation, also resulting in the hyperactivation of IL‐36 signals. Myeloperoxidase (MPO) deficiency results in the defective phagocytosis of neutrophils by monocytes, leading to the prolonged persistence of neutrophils in inflammatory skin lesions in GPP. Loss‐of‐function variants of *BTN3A3* lead to reduced IL‐36Ra activity and to excessive IL‐36 signaling, resulting in NFκB hyperactivation. In addition, the loss of function of BTN3A3 upregulates TNF‐α signaling, also resulting in NFκB hyperactivation and the increased secretion of cytokines/chemokines. Gain‐of‐function variants in *MEFV* induce pyrin inflammasome assembly, leading to caspase‐1 activation and to the increased secretion of IL‐1β and IL‐18, resulting in further inflammation.

There are seven genes that are currently known to be the genetic background of GPP—*IL36RN*, *CARD14*, *AP1S3*, *MPO*, *SERPINA3*,^11^ *BTN3A3*,^12^ and *MEFV*.^13^ This section will focus on the mechanisms, mainly autoinflammatory ones, whereby their variants are involved in the development of GPP (Table 1 and Figure 1).

**TABLE 1:** Genetic risk factors for generalized pustular psoriasis.

Disease‐associated gene	Disease‐associated molecule	Variant	Pathogenic mechanism and pathway
*IL36RN*	IL‐36 receptor antagonist (IL36Ra)	Loss‐of‐function variants	IL36Ra↓→IL36↑→NFκB↑ →IL‐36, IL‐8, TNF‐α, CXCL1, CXCL2, CCL20↑
*CARD14*	Caspase recruitment domain family member 14 (CARD14)	Gain‐of‐function variants	CARD14↑→NFκB↑→TNF‐α, IL‐1, IL‐36, IL‐8, CXCL1, CXCL2, CCL20↑
*AP1S3*	Adaptor‐related protein complex 1, sigma‐3 subunit (AP1S3)	Loss‐of‐function variants	AP1S3↓→AP1 complex↓ →endosomal translocation, autophagy↓ →p62 accumulation→NFκB↑ →IL‐36, IL‐8, IL‐1, TNF‐α, CXCL1, CXCL2, CCL20↑
*MPO*	Myeloperoxidase (MPO)	Loss‐of‐function variants	(1) MPO↓→neutrophil/monocyte proteases (CTSG, NE, PR3, CTSS)↑ →IL‐36↑ (2) MPO↓→formation of NETs↓ →neutrophil soluble proteases (CTSG, NE, PR3)↑→IL‐36↑ (3) MPO↓→efferocytosis of neutrophils↓ →delayed clearance of neutrophils in skin inflammatory lesions
*SERPINA3*	Serine protease inhibitor A3 (SERPINA3)	Loss‐of‐function variants	SERPINA3↓→CTSG↑ →IL‐36↑
*BTN3A3*	BTN3A3	Loss‐of‐function variants	(1) BTN3A3↓→IL36Ra↓→IL‐36↑ →MyD88↑→NFκB↑ →cytokines/chemokines↑ (2) BTN3A3↓→TNF‐α↑→NFκB↑ →cytokines/chemokines↑
*MEFV*	Pyrin	Gain‐of‐function variants	Pyrin↑→inflammasome assembly→caspase‐1 activation→ IL‐1β, IL‐18↑→NFκB↑ →neutrophil chemotactic factors (IL‐36γ, IL‐8, CCL20, CXCL1, CXCL2, etc.)↑

Abbreviations: CTSG, cathepsin G; CTSS, cathepsin S; NE, elastase; NETs, neutrophil extracellular traps; PR3, proteinase 3.

### 2.1 *IL36RN* loss‐of‐function variants

*IL36RN* variants are widely known as genetic defects associated with GPP.^11^, ^17^ Not only do individuals with biallelic *IL36RN* variants develop GPP, but so do those with monoallelic *IL36RN* variants.^18^, ^19^ Early‐onset GPP patients without preceding or coexisting psoriasis vulgaris frequently possess *IL36RN* variants.^18^, ^19^, ^20^, ^21^ It was reported that patients with GPP carrying *IL36RN* variants have more severe clinical features and a higher risk of systemic involvement.^22^

Loss of function of the anti‐inflammatory cytokine IL36Ra, which is encoded by *IL36RN*, induces the excessive signaling of IL‐1 cytokine family members and the increased production of inflammatory cytokines/chemokines and an exaggerated innate immune response, resulting in GPP pathogenesis.^2^, ^19^

### 2.2 *CARD14* gain‐of‐function variants

*CARD14* encodes caspase recruitment domain family member 14 (CARD14), and *CARD14* gain‐of‐function variants are related to both plaque psoriasis and GPP.^6^, ^23^, ^24^ Severe gain‐of‐function variants in *CARD14* give rise to GPP.^6^ Autosomal dominant familial cases of GPP caused by a gain‐of‐function variant in *CARD14* have been reported.^7^ However, patients with GPP due to *CARD14* variants represent a minority of GPP patients.^18^ Many GPP patients carrying *CARD14* variants also have plaque psoriasis.^6^ Furthermore, variants in *CARD14* are recognized to cause pityriasis rubra pilaris (PRP) type V^25^, ^26^ and CARD14‐associated papulosquamous eruptions.^27^

CARD14 is abundantly expressed in epidermal keratinocytes and predominantly works in those keratinocytes. As a scaffold protein, CARD14 has the function of activating NFκB. Gain‐of‐function variants in *CARD14* excessively activate the NFκB signaling pathway, leading to GPP‐pathogenic inflammation in the epidermis.^24^

### 2.3 *AP1S3* loss‐of‐function variants

*AP1S3* encodes adaptor‐related protein complex 1, sigma‐3 subunit (AP1S3), and *AP1S3* loss‐of‐function variants has been reported in patients with GPP.^8^

AP1S3 is mainly expressed in keratinocytes.^28^ AP1S3 is a component of a heterotetramer, adaptor‐related protein complex 1 (AP‐1), which transports various molecules from the trans‐Golgi network to the endosomes. In keratinocytes, *AP1S3* knockdown was reported to disturb the endosomal translocation of Toll‐like receptor 3 (TLR‐3), an innate pattern‐recognition receptor, and to interrupt the Toll‐like receptor signaling system.^8^ In addition, AP1S3 crucially works in the formation of autophagosomes from the trans‐Golgi network, especially in keratinocytes. AP1S3 deficiency was revealed to lead to the reduced function of autophagy and to abnormal p62 deposition in keratinocytes.^28^ p62 activates NFκB, and p62 deposition seems to cause the excessive activation of NFκB, resulting in accelerated IL‐1 signaling and the overexpression of IL‐36α.^28^

### 2.4 *MPO* loss‐of‐function variants

In 2020, Haskamp et al. studied 31 patients with GPP and found that *MPO* loss‐of‐function variants are GPP disease‐related variants.^9^ *MPO* codes the neutrophilic heme‐containing enzyme myeloperoxidase (*MPO*), and deficiency of *MPO* is considered to play some role in the pathogenesis of GPP.^9^

The allele frequency of *MPO* variants was significantly higher in a European GPP case cohort than in control subjects, a gnomAD dataset (32 264 non‐Finnish European genomes).^29^

Loss‐of‐function variants in *MPO* were reported to lead to MPO deficiency in the neutrophils/monocytes of GPP patients, and the activity of three neutrophil serine proteases (cathepsin G (CTSG), elastase (NE), and proteinase 3 (PR3)) was reported to be increased in MPO‐deficient neutrophils.^9^ The neutrophil serine proteases and the monocytic protease cathepsin S (CTSS) are thought to catalyze precursors of IL‐36, resulting in the formation of mature IL‐36. In this context, loss of function of MPO leads to elevated IL‐36 signaling. Furthermore, the production of neutrophil extracellular traps (NETs) decreases in neutrophils with defective *MPO* activity, leading to increases in soluble neutrophil proteases, which activate IL‐36 precursors more efficiently than NET‐bound proteases do.^9^ In addition, the efferocytosis of infiltrating neutrophils (i.e., the phagocytosis of neutrophils by monocytes) was defective in patients with defective MPO activity and in *MPO* ^−/−^ mice, resulting in the delayed clearance of neutrophils in skin inflammatory lesions.^9^ These data suggest that MPO plays important roles in the modulation of neutrophil‐related skin inflammation and that loss‐of‐function *MPO* variants provoke GPP through the upregulation of IL‐36 signals and the defective efferocytosis of neutrophils.^9^ A patient with mild GPP and a hypomorphic *MPO* variant has been reported, and the mild phenotype was suggested to be due to the incomplete hypomorphic loss‐of‐function *MPO* variant.^30^ In addition, the patient responded well to granulocyte and monocyte adsorption apheresis (GMA), and these findings suggest that GMA is effective for GPP patients with myeloperoxidase deficiency that results from *MPO* variants.^30^ A patient with a heterozygous hypomorphic variant of *MPO* was reported to have severe GPP that was refractory to spesolimab treatment.^31^

### 2.5 *SERPINA3* loss‐of‐function variants

Frey et al. identified a *SERPINA3* variant, c.966delT (p.Tyr322*), in two independent GPP patients, and they demonstrated that this variant was significantly associated with GPP.^10^ Theoretically, the protein product derived from the variant lacks the center loop, which is the active site of the protein. In addition, Frey et al. revealed that the variant causes nonsense‐mediated mRNA decay.^10^ From these data, they suggested that the loss‐of‐function variant in *SERPINA3* is a predisposing factor for GPP.^10^ *SERPINA3* codes serpin peptidase inhibitor clade A member 3 (SERPINA3).^10^ SERPINA3 inhibits a number of proteases, and it interacts strongly with CTSG. Neutrophilic serine proteases, including CTSG, NE, and PR3, are known to cleave IL‐36 precursors to mature, active forms, leading to the activation of the IL‐36 pathway. In this context, as SERPINA3 has inhibitory effects on CTSG, the loss‐of‐function variant in *SERPINA3* may reduce the inhibitory effect of SERPINA3 on the serine proteases and may result in the hyperactivation of IL‐36 cytokines, leading to GPP.^10^

### 2.6 *BTN3A3* loss‐of‐function variants

In 2023, Zhang et al. revealed that *BTN3A3* is significantly associated with GPP in Han Chinese patients, both in those with GPP alone and in those with GPP with psoriasis vulgaris, using gene burden testing.^12^ Sequencing of the entire coding regions of *BTN3A3* in 60 GPP patients and 100 control subjects showed one loss‐of‐function variant in *BTN3A3*, c.513G>A (p.Trp171Ter), to be significantly associated with GPP.^12^ BTN3A3, which is encoded by *BTN3A3*, is a transmembrane protein that is thought to work in signal transduction. The GPP‐associated *BTN3A3* variant leads to the loss of the B30.2 domain, a region significant for signal transduction,^32^ resulting in abnormal signal transduction. Functional analysis showed that the loss of function of BTN3A3 leads to the excessive activation of the TNF‐α‐mediated NF‐κB pathway and to the inhibition of IL‐36 receptor antagonist expression, leading to the upregulated production of inflammatory cytokines.^12^ Future research is awaited to determine how frequently BTN3A3 variants are involved in disease development for GPP patients in populations other than Han Chinese.

### 2.7 *MEFV* variants

MEFV variants are well‐known as a disease‐related factor for familial Mediterranean fever, a representative autoinflammatory disease/syndrome.^33^ *MEFV* products are known to control inflammatory pathways related to pyrin inflammasomes. A number of variants in *MEFV* are thought to induce neutrophil migration to tissues, resulting in inflammation.

Genomic DNA from 24 Japanese GPP patients was studied with next‐generation sequencing, and the allele frequencies of two *MEFV* variants, p.Arg202Gln and p.Ser503Cys, were found to be higher in GPP patients than in general controls.^13^ In the study, approximately 21% and 13% of Japanese GPP patients carried p.Arg202Gln and p.Ser503Cys variants in *MEFV*, respectively.

*MEFV* encodes pyrin, a regulator of innate immunity. Pyrin is mainly expressed in neutrophils and monocytes. Rho‐inactivating toxins and effectors from pathogenic bacteria activate caspase‐1 of pyrin inflammasomes, leading to accelerated IL‐1β and IL‐18 release.^34^ In GPP patients with disease‐related *MEFV* variants, the invasion of bacterial toxins and effectors into the skin might intensively activate neutrophils/monocytes via the excessive activation of pyrin inflammasomes.

The upregulated secretion of IL‐1β/IL‐18 from neutrophils carrying *MEFV* variants accelerates the secretion of neutrophil chemotactic factors, including IL‐36γ, IL‐8, CCL20, CXCL1, and CXCL2 from keratinocytes via NFκB signaling, leading to neutrophil chemotaxis and to inflammation, which forms pustules in GPP lesions.^13^

The fact that a variant of *MEFV*, a gene associated with familial Mediterranean fever, which is a typical autoinflammatory disease, is a predisposing factor for GPP is further evidence that GPP is an AiKD.

Therapies targeting inflammation signaling associated with *MEFV* might be promising treatment options for GPP patients harboring certain variants in *MEFV*.

## 3 COMPLEXITY OF THE GENETIC BACKGROUND OF GPP

After the identification of the seven GPP‐related genes (*IL36RN*, *CARD14*, *AP1S3*, *MPO*, *SERPINA3*, *BTN3A3*, and *MEFV*), a number of individuals were found to carry GPP risk alleles in more than one gene, such as risk alleles in both *IL36RN* and *MPO*,^9^ *IL36RN* and *AP1S3*,^28^ *MPO* and *AP1S3*,^29^ and *IL36RN* and *MEFV*.^13^ As additional GPP‐related genes are recognized, the number of GPP patients harboring variants in multiple GPP‐related genes is expected to grow further.^35^

In addition to variants in the seven GPP‐related genes mentioned above, variants in several other genes have been reported in GPP patients, including in *TNFAIP3*, *SERPINA1*, *TGFBR2, SERPINB3*, and *NFKB2*.^36^, ^37^, ^38^, ^39^, ^40^ Currently, all of these reports are at the level of case reports and do not have sufficient evidence to confirm these genes as GPP‐related. In addition, a single nucleotide polymorphism (SNP) in the 5′ untranslated region of *TNF* was reported to be associated with Han Chinese pediatric‐onset GPP,^41^ although future studies will be needed to confirm the effect of the *TNF* variant on TNF‐α expression and its relationship to the development of pediatric‐onset GPP.

In the future, in a significant proportion of patients with GPP, the nature of the disease might turn out to be polygenic. That is, in such cases GPP develops as a result of the accumulation of many small effects of a large number of relatively common genetic variants.

## 4 CURRENT AND NOVEL TREATMENTS FOR GPP THAT TARGET GENETIC BACKGROUND‐ASSOCIATED MOLECULES AND PATHWAYS

As mentioned above, our knowledge of the genetic predisposing factors and pathogenesis of GPP have recently expanded significantly. However, the therapeutic management of GPP remains challenging and involves much uncertainty.^1^

A number of biologics have been reported to be effective against GPP, but their relative merits have not yet been established.^42^

The recognition of genetic disease‐related factors and an accurate understanding of their involvement in the pathogenetic mechanisms of GPP are expected to contribute to development of efficient causal therapeutic strategies for GPP as an AiKD. In fact, various therapies targeting genetic predisposing factors and inflammatory pathways involved in the pathophysiology of GPP have been reported to be effective against GPP. These are described in the next section.

### 4.1 Treatments targeting IL‐36 signaling

It was demonstrated that the mRNA expression of *IL36RN* is lower in GPP patients with *IL36RN* variants and in those without *IL36RN* variants than in healthy controls, and the inflammatory conditions of GPP patients suggest that there is deficient *IL36RN* and unopposed IL‐36 signaling.^43^ The IL‐36 pathway is thought to be an important driver of inflammation in the GPP pathogenesis, whether or not the patient has *IL‐36RN* variants. The IL‐1/IL‐36‐chemokine‐neutrophil axis has been clarified as playing a crucial role in GPP pathogenesis not only in patients with IL36Ra deficiency, but also in patients with genetic predisposing factors other than *IL36RN* variants.^44^, ^45^, ^46^ In this context, inhibiting the IL‐36 axis is a promising therapeutic strategy for GPP.

Concerning the safety of inhibiting IL‐36 signaling, no impairment of immune function is seen in individuals with the loss of IL‐36 receptor function due to loss‐of‐function variants in *IL1RL2*, which encodes IL‐36 receptor IL‐1 receptor‐like 2 (IL1RL2).^47^ The findings imply that blocking the IL‐36 pathway would not significantly impair the host defense and could serve as a safe treatment strategy.^47^ A number of therapeutic candidates targeting the IL‐36 pathway are at various stages of research.^45^, ^48^, ^49^ Clinical trials have been conducted on two IL36 receptor antagonists, spesolimab (BI655130) and imsidolimab (ANB019), as the most promising treatments for GPP.^50^, ^51^, ^52^, ^53^

#### 4.1.1 Spesolimab

As for spesolimab, a phase I open‐label proof‐of‐concept study of a single intravenous injection (10 mg/kg) was performed on seven patients with GPP flare‐ups, and the rapid improvement of the flare‐ups was observed in all cases, with no serious adverse events.^50^ A randomized placebo‐controlled study of a single intravenous dose (900 mg) of spesolimab (the Effisayil 1 study) was conducted on GPP patients presenting GPP flares, and rapid and sustained improvements in GPP flare symptoms were achieved in most of the patients.^51^, ^52^ Furthermore, a multicenter, randomized, placebo‐controlled, phase IIb trial of subcutaneous spesolimab for the prevention of GPP flares (Effisayil 2) revealed that subcutaneous high‐dose spesolimab (600 mg loading dose followed by 300 mg every 4 weeks) significantly reduced the risk of GPP flares over 48 weeks in participating GPP patients with a history of at least two past GPP flares.^53^ In these clinical trials, interestingly, no obvious difference in the efficacy of spesolimab was seen between GPP patients with *IL36RN* variants versus those without such variants.^50^, ^52^, ^53^ The efficacy of spesolimab in patients without *IL36RN* variants further suggests that IL‐36 signaling plays an important role in the pathogenesis of GPP as an AiKD, even in GPP patients with genetic backgrounds other than *IL36RN* variants.

#### 4.1.2 Imsidolimab

Concerning imsidolimab, another IL‐36 receptor antagonist, a phase I clinical trial for healthy individuals showed a favorable safety profile (https://www2.anaptysbio.com/wp‐content/uploads/ANB019‐Phase‐1‐Study‐Poster‐EAACI‐2018.pdf). A phase II open‐label, single‐arm clinical trial of imsidolimab for GPP patients (the Gallop trial) was conducted.^54^ In the study, GPP patients received an intravenous dose of imsidolimab (750 mg) on the first day and three subcutaneous doses (100 mg) at 4, 8, and 12 weeks later, and the clinical responses were evaluated at weeks 4 and 16 following the first day.^54^ Six of the eight enrolled patients completed the study, and rapid and sustained improvements of the GPP symptoms and the pustular lesions were seen, with an acceptable safety profile, although one patient developed sepsis, possibly related to the study drug treatment.^54^ Thus, imsidolimab might be a promising treatment option for GPP.

### 4.2 Treatments targeting IL‐1 signaling and pyrin inflammasome signaling

Inhibitors of the IL‐1 pathway are used as a treatment for familial Mediterranean fever, where the excessive activation of pyrin inflammasome and IL‐1 signaling plays a major role in the pathophysiology.^55^ A variant of *MEFV*, which is the familial Mediterranean fever‐associated gene, has been revealed to be a genetic predisposing factor for GPP,^13^ and it is possible that treatments for familial Mediterranean fever, including colchicine and IL‐1 signal blocking agents, may hold promise as therapeutic agents for GPP. The IL‐1 signal blocking agents anakinra and canakinumab are in clinical use for pyrin‐associated autoinflammatory syndromes, familial Mediterranean fever, and hyper IgD syndrome/mevalonate kinase deficiency.^55^ Anakinra has also been reported to be effective against GPP.^56^ In addition, gevokizumab, another IL‐1 receptor antagonist, was reported to be partially effective in two patients with GPP.^57^ A patient with GPP was reported to benefit from treatment with canakinumab, a monoclonal anti‐IL‐1β antibody.^58^ Based on case reports on the efficacy of these therapies that target IL‐1 signaling, it is expected that IL‐1 inhibitors may be effective against GPP, which is an AiKD, although at present there is insufficient clinical data to recommend treatments targeting IL‐1 signaling.^56^, ^59^

Furthermore, pyrin inflammasome signaling inhibitors may be potential treatments for GPP in the future.

## 5 OUTLOOK

GPP‐associated variants in a number of genes (*IL36RN*, *CARD14*, *AP1S3*, *MPO*, *SERPINA3*, *BTN3A3*, and *MEFV*) have been recognized. From the information on genetic predisposing factors for GPP, we have obtained a novel understanding of the pathogenesis of GPP as being mainly associated with a number of molecules involved in the transduction of inflammatory signals and a variety of cytokine pathways, particularly the IL‐36 pathway. Recently acquired insights into the molecular pathogenesis of GPP verify the notion that GPP is a representative AiKD, and this verification may pave the way for novel therapeutic approaches that target innate immune pathways in GPP. Our updated knowledge of significant inflammation pathways, mainly those involving innate immune responses, in GPP pathophysiology highlights the idea that molecular targeting agents to inhibit cytokine pathways such as IL‐36 signaling are promising approaches for ameliorating quality of life in GPP patients. In the near future, it is expected that accurate information about the pathogenesis and genetic background of GPP will contribute to the establishment of precision medicine and personalized treatment for patients with this disease.

## CONFLICT OF INTEREST STATEMENT

The author declares no conflict of interests for this article. Masashi Akiyama is an Editorial Board member of the *Journal of Dermatology* and the author of this article. To minimize bias, he was excluded from all editorial decision‐making related to the acceptance of this article for publication.
